# Effects of auricular vagal neuromodulation therapy combined with slow-paced diaphragmatic breathing in individuals with postural tachycardia syndrome: a randomised controlled trial protocol

**DOI:** 10.1136/bmjopen-2026-120159

**Published:** 2026-06-24

**Authors:** Tobias Brandl, Florian Pichler, Igor Grabovac, Thomas Waldhoer, Hans Keller, Dorothee Fenneker, Alexander Niessner, Ali Kapan

**Affiliations:** 1 Center for Public Health, Department of Social and Preventive Medicine, Medical University of Vienna, Vienna, Austria; 2 Center for Public Health, Department of Epidemiology, Medical University of Vienna, Vienna, Austria; 3 Vienna Healthcare Group, 2nd Department of Medicine with Cardiology and Intensive Care Medicine, Clinic Landstrasse, Vienna, Austria

**Keywords:** Cardiovascular Disease, Clinical Trial, NEUROLOGY, Chronic Disease

## Abstract

**Introduction:**

Postural orthostatic tachycardia syndrome (POTS) is a multifactorial disorder of the autonomic nervous system characterised by an excessive increase in heart rate (HR) on standing and a wide range of debilitating symptoms, including fatigue, exercise intolerance, cognitive impairment and a high prevalence of depression, anxiety and sleep disturbances. Conventional pharmacological strategies often offer limited relief and do not sufficiently address non-cardiac symptoms. Auricular vagal neuromodulation therapy (AVNT) and slow-paced diaphragmatic breathing (SDB) have each demonstrated promise in modulating autonomic function and alleviating symptom burden but their combined effects in POTS have yet to be investigated.

**Methods and analysis:**

This single-centre, partially blinded, 12-week randomised controlled trial will be conducted at the Medical University of Vienna. A total of 100 participants with confirmed POTS will be recruited, with 25 per group. This sample size is already accounting for an anticipated 30% dropout rate. Participants will be randomised using stratified block randomisation with a 1:1:1:1 allocation ratio, stratified based on the presence or absence of post-exertional malaise (yes/no) to one of four groups: (1) AVNT+SDB, (2) AVNT+normal breathing, (3) sham AVNT+SDB and (4) sham AVNT+normal breathing. The primary endpoint is the change in orthostatic HR (ΔHR) during a 70° head-up tilt test. Secondary outcomes include beat-to-beat blood pressure responses, respiratory sinus arrhythmia, end-tidal CO₂, handgrip strength, activity monitoring and validated patient-reported measures, including Chalder Fatigue Scale, Malmö POTS Symptom Score, Nijmegen Questionnaire, Short Form Health Survey, Hospital Anxiety and Depression Scale and Vanderbilt Orthostatic Symptom Score. Interventions consist of daily 60-minute AVNT (or sham) sessions combined with standardised breathing training (10–15 min/day).

**Ethics and dissemination:**

This study has been approved by the Ethics Committee of the Medical University of Vienna (EK number 1270/2024) and will be conducted in accordance with the Declaration of Helsinki and International Conference on Harmonisation-Good Clinical Practice guidelines. Results will be disseminated through peer-reviewed publications, conference presentations and reporting in the trial registry. Participants will receive study results in an accessible format.

**Trial registration number:**

NCT06996314.

**STRENGTHS AND LIMITATIONS OF THIS STUDY**

- This is, to our knowledge, the first randomised controlled trial to investigate the combined effects of auricular vagal neuromodulation therapy and slow-paced diaphragmatic breathing in postural orthostatic tachycardia syndrome.
- The 2×2 factorial design allows evaluation of both individual and combined intervention effects. The study includes objective autonomic measurements alongside validated patient-reported outcomes.
- The single-centre design may limit generalisability of the findings. Blinding is not feasible for the breathing intervention, which may introduce performance bias.

## Introduction

###  Background and rationale

Postural orthostatic tachycardia syndrome (POTS) is a complex disorder of the autonomic nervous system, manifesting as a substantial heart rate (HR) increment of over 30 beats per minute on transitioning to an upright position, without an accompanying decrease in blood pressure (BP).^1^ The underlying pathophysiological theories encompass autoimmune and auto-inflammatory responses,^2^ alongside small fibre peripheral and autonomic neuropathy, hyperadrenergic states and hypovolaemia.^3 4^

POTS can be triggered by biological, psychological and social factors, such as viral infections, vaccinations, pregnancy, surgical procedures, physical trauma and psychosocial stressors.^2 5^ POTS markedly impairs both physical and cognitive abilities, with patients suffering from chronic fatigue, dizziness and exercise intolerance that cause decreased daily activity and worsening deconditioning.^6^ Cognitive symptoms, such as ‘brain fog’, difficulties with concentration and memory issues, arise from autonomic dysfunction and decreased cerebral blood flow.^7 8^ These challenges are intensified by high rates of depression (87%), sleep disturbances (over 50%) and anxiety, forming a complex symptom burden that further worsens the condition.^9 10^ Epidemiologically, POTS affects an estimated 0.2% to 1.0% of the population in developed nations, with a notable predominance among females (with a female to male ratio of 5:1). Challenges in the clinical recognition of POTS often lead to misdiagnoses and delays in accurate diagnosis, highlighting the need for increased awareness and understanding of this condition.^11 12^

While a 2018 systematic review highlighted limited evidence for effective management,^13^ more recent analyses support integrated approaches that combine pharmacological and non-pharmacological strategies.^14^ Traditional pharmacological treatments aim to reduce excessive HR through volume expansion (hydration, sodium supplementation, fludrocortisone), venous pooling reduction (compression garments, midodrine), parasympathetic enhancement (cholinesterase inhibitors), sympathetic modulation (β-blockers, α2-agonists) and direct HR control (ivabradine).^15^ However, these approaches often provide limited relief and are linked to both cardiac and non-cardiac side effects, failing to effectively address non-cardiac symptoms.^16 17^

Although there have been limited studies examining the impact of Auricular Vagal Neuromodulation Therapy (AVNT) on POTS, emerging evidence from case reports and clinical trials indicates its potential.^18 19^ Notably, a recent double-blind, sham-controlled trial study^20^ revealed that daily AVNT for 2 months notably enhances postural tachycardia and autonomic symptoms, indicating improved autonomic control and vascular function without affecting BP. AVNT has also been associated with improved autonomic regulation and reduced inflammatory markers, highlighting its therapeutic potential in POTS. Further research is needed to explore its combination with other treatments and to optimise stimulation parameters. Respiratory-gated AVNT, particularly during the expiratory phase, may further enhance its effects due to physiological variations in vagal activity during breathing.^21^ In addition to AVNT, studies suggest that 80%–90% of POTS patients experience dysfunctional breathing (DB), with up to 65% experiencing severe breathlessness even at rest.^22^ Therefore, the management of DB has emerged as another important aspect of POTS management. DB manifests as a variety of respiratory abnormalities, including hyperventilation, periodic deep sighs, thoracic-dominated breathing, forced abdominal expiration and thoracoabdominal asynchrony.^23^ Although DB is considered a symptom rather than a cause of POTS, its prevalence makes it an important target for intervention. Because breathing, unlike many other autonomic functions, can be consciously perceived and controlled, it offers a unique opportunity for behavioural interventions that target the autonomic nervous system.^24^ Specifically, incorporating slow-paced diaphragmatic breathing (SDB) at 0.1 Hz has been shown to enhance vagal activity.^25^ This suggests that combining slow-paced breathing techniques with AVNT could potentially optimise therapeutic efficacy for POTS patients.

However, despite the individual promise of both AVNT and breathing techniques, their combined effects in POTS have not yet been investigated. To address this gap, we propose a randomised controlled trial (RCT) to evaluate the efficacy and feasibility of an integrated approach combining AVNT with slow-paced breathing. This study aims to explore potential synergistic effects and improve symptom management in patients with POTS. Both interventions have favourable safety profiles; transcutaneous auricular vagus stimulation is generally associated with mild, transient local effects, while SDB may occasionally cause light-headedness if performed incorrectly. These risks will be minimised through standardised instruction and monitoring. A 2×2 factorial design (active vs sham AVNT; SDB vs normal breathing (NB)) was selected to assess both individual and combined effects, while maintaining statistical efficiency. Sham AVNT controls for device-related effects, and normal breathing isolates the specific contribution of slow-paced breathing.

### Objectives

The main aim is to assess whether 12 weeks of AVNT combined with SDB (AVNT+SDB) produces a greater improvement in autonomic function, measured as a reduction in the orthostatic HR change (ΔHR) during a standardised 70° head-up tilt, than AVNT+NB, sham AVNT+SDB or sham AVNT+NB. ΔHR is predefined as the mean HR during the 10-minute upright head-up tilt phase minus the mean HR during the 10-minute supine baseline period; comparisons between AVNT+SDB and each other group are confirmatory, while all other pairwise comparisons are exploratory.

Secondary objectives include characterising between-group differences in beat-to-beat BP responses (mean arterial pressure (ΔMAP), systolic BP (ΔSBP), diastolic BP (ΔDBP), the initial orthostatic nadir, and time to recovery). Respiratory sinus arrhythmia (RSA) is assessed as an index of cardiac vagal modulation using ECG-derived HR variability (HRV), assessing patient-reported outcomes using the Short Form Health Survey (SF-36) for health-related quality of life, the Hospital Anxiety and Depression Scale (HADS) for symptoms of anxiety and depression, Chalder Fatigue Scale (CFS), the Malmö POTS Symptom Score (MAPS) and the Nijmegen Questionnaire (NQ) for symptoms of dysfunctional breathing.

As a safety objective, the trial will evaluate tolerability and systematically record adverse events (AEs) and serious AEs (SAEs), including treatment-related discontinuations, throughout the study, with descriptive comparisons of event profiles across the four groups.

## Methods

### Patient and public involvement

Patients or members of the public were not actively involved in designing this trial. However, feedback from patient support groups such as Long COVID Austria and ME/CFS Austria helped inform the recruitment strategy. Additionally, a qualitative substudy is planned to collect systematic feedback from involved patients regarding their experiences with the intervention and the trial procedures. This input will be considered when interpreting results and shaping the dissemination strategy.

### Trial design

This study is an ongoing single-centre, partially blinded, 12-week RCT with a sham control conducted at the Medical University of Vienna. Participant enrolment began on 19 March 2026. This protocol was developed in accordance with the Standard Protocol Items: Recommendations for Interventional Trials (SPIRIT) guidelines. The study is registered at ClinicalTrials.gov (Identifier: NCT06996314) and will be reported according to the Consolidated Standards of Reporting Trials (CONSORT) statement.^26^

Participants will be randomised using stratified block randomisation in a 1:1:1:1 ratio to one of four groups:

- AVNT+SDB
- AVNT+NB
- Sham AVNT+SDB
- Sham AVNT+NB

The randomisation will be based on the presence or absence of post-exertional malaise (PEM) (yes/no).

### Trial setting

The trial will be conducted at the Medical University of Vienna, in cooperation with the second Department of Medicine with Cardiology and Intensive Care Medicine in a specialised autonomic laboratory.

### Eligibility criteria

#### Inclusion criteria

- Age between 18 and 65 years.
- Formal POTS diagnosis confirmed by current consensus criteria: sustained HR increase of at least 30 bpm within 10 min of standing (head-up tilt test (HUTT), 70°), in the absence of orthostatic hypotension (SBP fall ≥20 mm Hg or diastolic BP fall ≥10 mm Hg), with characteristic symptoms persisting for at least 6 months.^27^
- Participants with Long COVID will be included if they meet the formal diagnostic criteria for POTS. Among those experiencing PEM, only individuals who have been clinically stable for more than 2 months, exhibit mild PEM and regularly employ pacing strategies will be eligible.
- Participants on POTS medication must be on stable dosing for at least 4 weeks prior to enrolment. Medication will be continued throughout the study, and participants will be grouped by medication class for subgroup analysis. Any changes in medication during the study must be reported immediately and documented for analysis.
- Participants must be able and willing to provide written informed consent.
- Sufficient German or English language proficiency to complete questionnaires and provide informed consent.

#### Screening of participants without a prior diagnosis

Individuals without a prior formal POTS diagnosis but who report characteristic symptoms for at least 6 months (eg, dizziness, chronic fatigue, syncope, brain fog, palpitations, exercise intolerance) will undergo a standardised HUTT (70°) at the study site in order to establish a formal diagnosis according to current consensus criteria.

- HR and BP will be recorded during a supine rest period and during head-up tilt. The orthostatic HR response is assessed as the change in HR from supine to upright position (ΔHR) within the first 10 min of tilt.
- A ΔHR of ≥30 bpm within 10 min of head-up tilt (70°), in the absence of orthostatic hypotension (SBP fall ≥20 mm Hg or diastolic BP fall ≥10 mm Hg), fulfils the diagnostic criteria for POTS; in participants with a ΔHR of 25–29 bpm, the diagnostic criteria are likewise considered fulfilled if typical orthostatic symptoms are present and clinical judgement supports the diagnosis.

Participants who do not meet the diagnostic criteria will not be enrolled.

#### Exclusion criteria

Participants meeting any of the following criteria will be excluded from the study:

- Significant hypertension, either in the supine or standing position, defined as BP readings exceeding 150 mm Hg SBP or 100 mm Hg DBP.
- Orthostatic hypotension, defined as a consistent BP decrease of >20 mm Hg SBP or >10 mm Hg DBP within 10 min of standing.
- Cardiac rhythm or conduction disorders that may interfere with autonomic assessment, including atrial fibrillation (paroxysmal, persistent or permanent), presence of a pacemaker or an implanted cardioverter defibrillator.
- Recent stroke or myocardial infarction within the past 6 months.
- History or presence of significant systemic diseases, including malignancies, autoimmune disorders, clinically relevant immunological or haematological diseases or severe anaemia (haematocrit <28%).
- Neurological conditions that may confound autonomic or cardiovascular measurements, including seizure disorders, Menière’s disease or a surgically severed vagus nerve (eg, prior vagotomy).
- Orthopaedic, rheumatological or postoperative conditions that limit safe participation in HUTT.
- Current pregnancy or lactation.
- Ear-related contraindications to auricular stimulation, including cochlear implants, history of ear surgery, recent ear injuries or infections, or negative prior experiences with electrotherapy.
- Acute illness, including fever at the time of assessment.
- Current psychiatric disorders that, in the judgement of the investigators, may interfere with study participation or adherence.
- Inability or unwillingness to provide written informed consent.

Insufficient German or English language proficiency to understand study procedures, provide informed consent, or complete study questionnaires.

### Eligibility criteria for sites and individuals

Since this is a single-centre trial conducted at the Medical University of Vienna, no additional site-specific eligibility criteria are necessary. The trial will take place in the autonomic laboratory of the second Department of Medicine with Cardiology and Intensive Care Medicine at Clinic Landstrasse. Assessments, including physiological testing and data collection, will be performed by a trained study nurse with expertise in autonomic and cardiovascular assessments. The breathing therapy intervention will be administered by a licensed physiotherapist with over ten years of experience in respiratory and autonomic rehabilitation.

### Intervention and comparator

Before starting the intervention, all participants attend a 60-minute introductory training session led by a physiotherapist experienced in respiratory and autonomic therapy, where the correct use of the device, electrode placement and adherence strategies are covered. The first stimulation session is carried out under supervision to ensure proper application and participant safety.

#### Auricular vagal neuromodulation therapy

AVNT will be administered using the Nurosym device (Parasym Health, London, UK), a CE-certified and clinically validated medical device. Stimulation is applied via ear electrodes placed at the cymba conchae of the left ear; an anatomical site innervated by the auricular branch of the vagus nerve. The device settings are uniformly applied to all participants according to established clinical standards and in accordance with the study by Stavrakis *et al*.^20^ Specifically, stimulation is administered with a pulse frequency of 20 Hz and a pulse width of 200 microseconds. The intensity is individually adjusted for each participant to evoke a noticeable but comfortable tingling sensation without causing pain or discomfort. Each participant undergoes one stimulation session daily, lasting 60 min, over a total intervention period of 12 weeks.

#### Sham stimulation

Participants in the sham groups use the same device with the same electrode placement. However, the sham device does not deliver electrical current. Instead, a sound-sham method is used, in which the device emits pulsed acoustic signals that mimic the auditory cues of active AVNT but have no physiological effect on vagal pathways. This method provides a high-quality placebo control and preserves participant blinding.

#### Diaphragmatic breathing

Diaphragmatic breathing therapy will be carried out according to a standardised protocol with individual adjustments based on baseline assessment using the Breathing Pattern Assessment Tool (BPAT). This will enable differentiation between restrictive and functional breathing disorders. Resting respiratory rate will also be documented to establish individual baseline values and guide therapy progression.

The technique involves nasal inhalation with controlled abdominal expansion followed by passive exhalation through lightly pursed lips. When needed, segmented inhalations and brief inspiratory pauses will be included to improve tidal volume control and muscle coordination. Therapy will progress gradually, with the respiratory rate decreased by about 20%–30% relative to baseline during the initial phase (aiming for 10–11 breaths/min in weeks 1–2; 8–9 breaths/min in weeks 3–4). From week 4 onwards, participants will switch to a slow-breathing protocol of 4 s inhalation, 6 s exhalation and a 2–3 s postexhalation pause, targeting roughly 5 breaths/min.

Breathing therapy will be supported by a mobile application that provides visual or acoustic pacing cues and records practice frequency. The planned therapy duration will be at least 10 min daily during weeks 1–4 and at least 15 min per day from week five onwards. Safety monitoring will require immediate cessation of the exercise and notification of the study team in cases of dizziness, paraesthesia, thoracic discomfort or presyncope. Participants will submit a weekly breath training diary via the breathing app to allow monitoring of adherence to the breathing intervention.

#### Criteria for discontinuing or modifying the intervention

Participation in the intervention may be discontinued or modified if participants experience side effects that make continuation unreasonable. Such side effects include local skin irritation at the electrode site, pain or severe discomfort during stimulation, pronounced dizziness, syncope, chest discomfort or nausea. Pregnancy during the study period is also an exclusion criterion. In addition, participants may withdraw from the intervention at any time on request, without disadvantage. All modifications or withdrawals will be systematically documented and included in the analysis.

#### Strategies to improve adherence

Adherence is maintained through a multistep process. At the start of the study, participants receive thorough training from a physiotherapist with over 10 years of clinical experience in respiratory and autonomic therapy. This training covers detailed instructions on correct device use, electrode placement and performing the breathing exercises. During the study, daily usage of AVNT and breathing training is logged via a mobile application that records usage time and frequency. Weekly adherence questionnaires are gathered to document how often, how long and how well the interventions are tolerated. Regular follow-ups (every 2–3 weeks), either by phone or in person, are conducted to help keep motivation high, address concerns and support compliance.

#### Concomitant care

Existing pharmacological treatments for POTS (such as β-blockers, ivabradine, midodrine or fludrocortisone) may be continued throughout the 12-week intervention period, provided that the medication regimen has been stable for at least 4 weeks prior to enrolment. Changes in medication during the intervention phase should be avoided whenever possible. If medication adjustments are medically necessary, these changes will be documented and considered in appropriate subgroup or sensitivity analyses. Concomitant therapies that directly influence autonomic function (eg, additional vagal stimulation methods, newly initiated breathing techniques or newly started autonomic-modulating medications) are not permitted during the intervention period. Supportive non-pharmacological measures, such as increased fluid intake, higher salt consumption or the use of compression garments, are allowed, as they reflect standard care for individuals with POTS.

### Outcomes

Prior to each study assessment, participants will be instructed to arrive in a fasting state and to abstain from caffeine, alcohol, nicotine and other stimulants for at least 12 hours before the visit. Whenever clinically safe and feasible, medications with known cardiovascular effects will be temporarily withheld prior to study assessments. For most POTS-related medications, a withholding period of at least 48 hours prior to testing will be applied. In participants receiving β-blocker therapy, particularly at higher doses, a gradual dose reduction over up to 72 hours prior to assessment is recommended, depending on the specific agent and dosage. All medication adjustments and temporary withholding procedures will be performed in consultation with the treating physician. Following completion of the respective study assessments, participants will resume their usual prescribed medication regimen unchanged for the remainder of the intervention period.

#### Procedure

On arrival at the study site, participants are welcomed, informed about the procedure and prepared for the examination.

1. Handgrip strength assessment
  The examination begins with a handgrip strength assessment performed in the seated position (see outcomes section for details). After completion of the handgrip test, participants rest briefly before further instrumentation.
2. Simultaneous application of all monitoring devices
  Next, all monitoring and measurement devices are applied simultaneously before the autonomic assessment begins. Continuous monitoring includes:

- 12-lead ECG for continuous HR and rhythm assessment
- Non-invasive beat-to-beat BP monitoring using a finger-cuff system
- Sidestream capnography via nasal cannula for continuous end-tidal CO₂ (EtCO₂) measurement
- Pulse oximetry attached to the left index finger for oxygen saturation

1. Supine phase—autonomic assessment (approximately 20 min total)
  The examination begins with a brief calibration phase of 2–3 min, during which all monitoring devices are calibrated and signals optimised, including finger-cuff BP system calibration.
  Once stable signals are confirmed, RSA is assessed using a standardised paced-breathing protocol. Participants are first familiarised with the breathing technique for 1 min using a visual pacer, then asked to maintain a breathing rate of 6 breaths per minute (0.1 Hz: 5 s inhalation, 5 s exhalation) for 2 min while ECG, beat-to-beat BP and EtCO₂ are recorded continuously. After completing the RSA assessment, participants return to spontaneous breathing and rest for 3–5 min to allow cardiovascular parameters to stabilise. Following this recovery period, a 10 min baseline recording is obtained while participants breathe spontaneously and remain still. This entire 10 min baseline phase is used for analysis to ensure optimal signal stability before proceeding to the tilt phase.
  Throughout the entire supine phase, HR (ECG), beat-to-beat BP and EtCO₂ are recorded continuously. Event markers are set to delineate the different assessment phases for subsequent analysis.
2. Upright phase HUTT (15 min or until termination)
  Following the supine baseline, the tilt table is moved from horizontal to 70° within a few seconds and maintained in the upright position for 15 min or until intolerance occurs. Throughout the tilt phase, all haemodynamic parameters (ECG, beat-to-beat BP, EtCO₂, SpO₂) are recorded continuously. Symptoms will be documented using the Vanderbilt Orthostatic Symptom Score (VOSS),^28^ which evaluates nine orthostatic symptoms on a scale of 0–10.
  The VOSS is administered at the 10-minute mark or immediately on test termination. Additionally, the examiner continuously documents the type, onset time, and severity (0–10) of any newly occurring symptoms throughout the upright phase. The test is terminated early in case of syncope, presyncope, severe symptomatic hypotension, participant request or other medical concerns.

#### Primary endpoint

Our primary endpoint is the orthostatic HR change (ΔHR), derived from continuous 12-lead ECG (R-R intervals) throughout the protocol using the CNSystems Task Force Monitor (CNSystems Medizintechnik, Graz, Austria). ΔHR is defined as the mean HR during the 10-minute upright head-up tilt phase minus the mean HR during the 10-minute supine baseline period.

**ΔHR=mean HR during the 10-minute upright phase−mean HR during the 10-minute supine baseline**

#### Secondary outcomes

BP: Continuous beat-to-beat BP readings will be obtained using a non-invasive finger-cuff system and summarised into 1-minute averages. Additionally, brachial oscillometric BP measurements will be performed at baseline and every 3–5 min for calibration and verification. The outcomes include changes in ΔMAP, ΔSBP and ΔDBP, defined as the mean values during the 10-minute upright phase minus the mean values during the 10-minute supine baseline period. Additional outcomes include the presence of orthostatic hypotension according to predefined thresholds and initial orthostatic responses, such as the nadir SBP within the first 30 s after tilt-up and the time to recovery.

RSA: RSA is quantified as an index of cardiovagal parasympathetic function using the CNSystems Task Force Monitor (CNSystems Medizintechnik, Graz, Austria). RSA amplitude (ΔHR) is calculated using the peak-to-valley method, which determines the difference between maximum HR during inspiration and minimum HR during expiration for each breathing cycle, then averaged across all cycles during the 2-minute paced-breathing period, and expressed in beats per minute (bpm). Values are interpreted considering established age-related and sex-related variations in RSA.^29^

Breathing assessments: Symptoms of dysfunctional breathing will be assessed using the NQ, a validated 16-item self-report instrument. Items are rated on a 5-point Likert scale (0–4), yielding a total score of 0–64, with higher scores indicating greater symptom burden; scores ≥23 suggest clinically relevant hyperventilation syndrome. The NQ is widely used as a screening tool in clinical populations.^30^ In addition to self-report, breathing patterns will be assessed using the BPAT, a validated observational tool. A trained assessor will evaluate key features of dysfunctional breathing at rest, including thoracic versus abdominal movement, chest wall symmetry and use of accessory muscles. Items are scored based on severity and summed to provide an overall index of breathing pattern disturbance.

The BPAT has been shown to be a reliable clinical tool for identifying dysfunctional breathing patterns and is suitable for use in both research and clinical practice.^31^

Capnography: End-tidal carbon dioxide (EtCO₂) will be continuously monitored using a sidestream capnograph connected to a nasal cannula with an integrated sampling line. Measurements will be taken throughout the supine baseline period and during the upright tilt phase, with recordings synchronised with ECG and BP data. EtCO₂ values will be collected on a breath-by-breath basis and stored for offline analysis.^32^

Handgrip strength performance: Before and after the HUTT, participants will undergo a handgrip strength assessment using a CAMRY Digital Hand Dynamometer while seated. To evaluate muscle strength, fatigue and recovery capacity, a validated repetitive isometric handgrip strength protocol will be applied. Each participant will complete two rounds of a 10×3 s maximal isometric contraction test, separated by 1 hour. Within each round, participants will perform ten maximal contractions of 3 s each, with 5 s of rest between trials, paced by an interval timer. All measurements will be conducted in a seated position with the forearm supported and the hand in a neutral position.^33^

From this protocol, the following parameters will be calculated:

- Maximum force (Fmax): The highest value of the 10 repetitions per round.
- Mean force (Fmean): Average of all 10 repetitions per round.
- Fatigue ratio: Fmax/Fmean of round 1. Higher values indicate greater force decline within a session (ie, higher fatigability).
- Recovery ratio: Fmean (round 2)/Fmean (round 1). Higher values reflect better recovery capacity between rounds.

This method allows for a more sensitive and objective evaluation of physical fatigue and recovery potential in older adults, beyond conventional HGS testing.

Activity and HR tracker: A wearable device (Fibion Flash) will monitor activity levels and HR for 3 days (2 nights) via ECG long-term adhesive electrodes at two points during the study: once in the first week and again after the 10th week of the intervention. This device provides a non-invasive, objective and real-time data collection method that tracks these metrics over an extended period. While the data are continuously collected and synced to a dedicated app or online portal, participants will not have access to real-time data such as HR, sleep, or activity levels to avoid influencing their behaviour. The collected data will be analysed by the research team.

Questionnaire management: Following the physiological measurements, participants complete the questionnaires either on-site (depending on the patient’s condition) or online at home.

MAPS: A German translation of the MAPS questionnaire, previously used in the study by Stick *et al*^34^ and used here with permission, will be employed in this trial. This questionnaire-based system helps individuals with POTS self-assess their symptom burden. It uses a visual analogue scale from 0 (no symptoms) to 10 (severe symptoms) to measure the severity of 12 common symptoms experienced over the past week. The total score can reach up to 120 points, providing a comprehensive assessment of symptom severity in POTS patients. Based on empirical data and clinical expertise, this scoring system allows for the systematic assessment and monitoring of the impact of POTS on patients’ daily lives by both patients and healthcare providers.^35^

SF-12: The validated German version of the SF-12 will be used. This 12-item patient-reported questionnaire assesses patient health across eight dimensions, including vitality, pain and general health perceptions. Each scale is converted to a 0–100 scale, with higher scores indicating better health and quality of life.^36^

HADS: The validated German version of the HADS (HADS-D) will be used. The HADS is a 14-item self-report questionnaire consisting of two 7-item subscales that evaluate anxiety and depression.^37^

Chalder Fatigue Questionnaire (CFQ): Fatigue severity will be assessed using the CFQ, a validated self-report questionnaire designed to measure physical and mental aspects of fatigue. The CFQ has demonstrated good reliability and validity in both clinical and community populations and will be used to quantify subjective fatigue severity at the time of assessment.

NQ: Symptoms of dysfunctional breathing will be assessed using the NQ, a validated 16-item self-report instrument. The questionnaire has demonstrated high sensitivity and specificity for distinguishing individuals with hyperventilation-related symptoms from healthy controls and is suitable as a screening tool in clinical and research settings.^38^

AEs: Participants will be encouraged to report any new or unusual symptoms to the study team promptly. This could be done via phone, email or during routine check-ins. These events, regardless of whether or not they are related to the study, will be recorded and monitored by the team. Discontinuation due to AEs: If a participant chooses to discontinue the intervention due to a perceived decline in health or adverse reactions, this will be documented and analysed. To ensure accurate and consistent data, participants might be asked to complete a simple AE questionnaire on a weekly basis. This could be administered as an online survey or over the phone, depending on the participant’s preference. If any severe or concerning symptoms arise, participants will be instructed to contact the study team immediately. All collected data will be systematically recorded and evaluated to monitor the safety of the intervention.

### Harms

**Figure 1:**
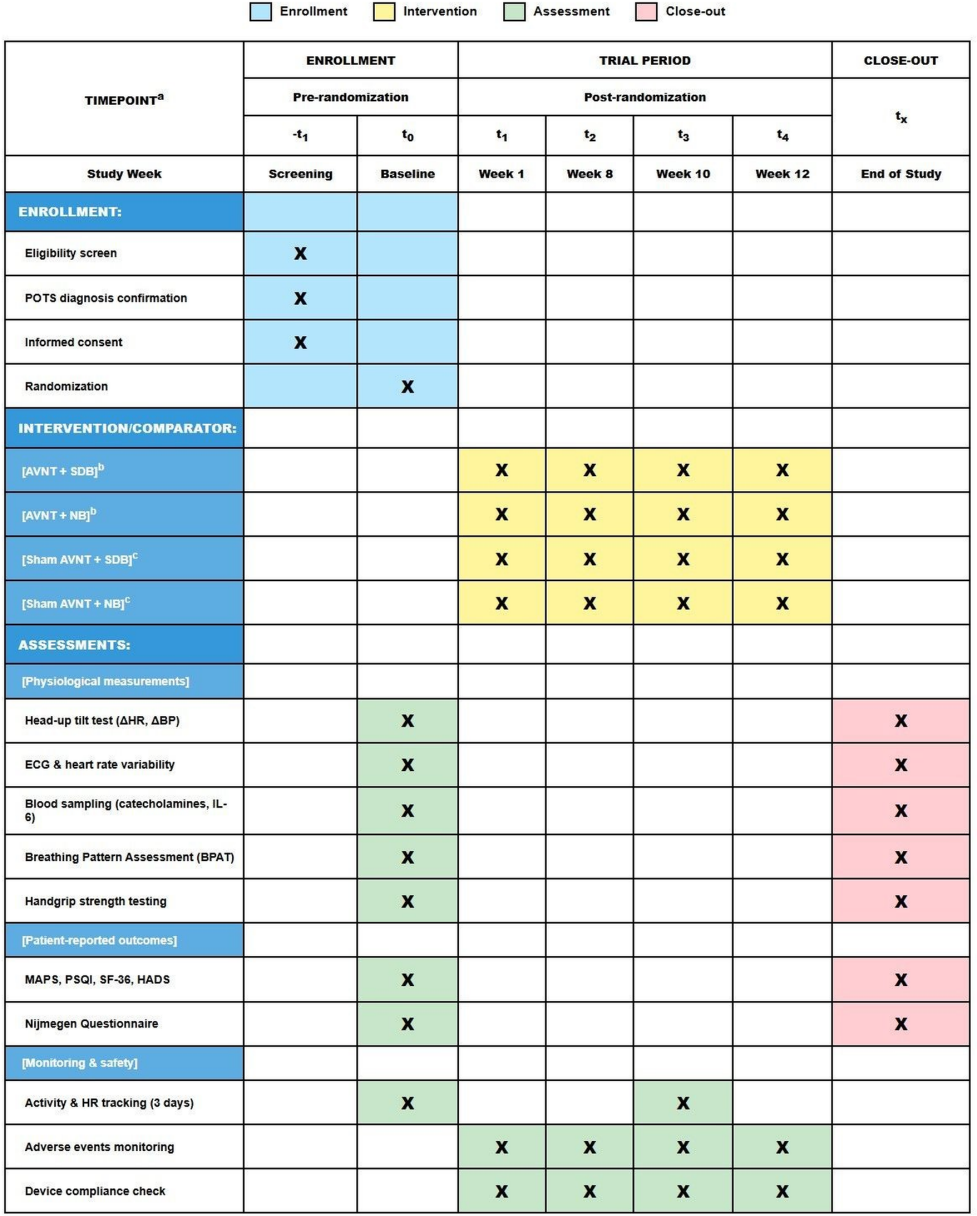
Study design, assessment schedule, and intervention procedures.Participant timeline. ^a^List target time points and acceptable time windows in this row (eg, ±3 days). ^b^Active AVNT (Auricular Vagal Neuromodulation Therapy): 60 min daily using Nurosym device; SDB (slow deep breathing): 4 s inhalation, 6 s exhalation, 2–3 s pause; NB (normal breathing): spontaneous breathing pattern. ^c^Sham AVNT uses a sound-sham method with the same device placement but no electrical stimulation. Primary endpoint: orthostatic HR change (ΔHR−Delta HR)=mean HR during the 10-minute upright phase minus mean HR during the 10-minute supine baseline period. Medication washout: cardiovascular medications (beta-blockers, ivabradine, midodrine) discontinued 48 hours prior to physiological assessments. Study duration: 12 weeks total intervention period. Close-out visit=final endpoint measurements. BP, blood pressure; BPAT, Breathing Pattern Assessment Tool; HADS, Hospital Anxiety and Depression Scale; HR, heart rate; MAPS, Malmö POTS Symptom Score; POTS, Postural Orthostatic Tachycardia Syndrome; SF-12, Short Form Health Survey, PSQI, Pittsburgh Sleep Quality Index.

Harms are defined as any AEs or side effects occurring during the trial, regardless of their causal link to the intervention. They will be assessed systematically through weekly AE questionnaires, supplemented by spontaneous reports from participants via phone, email or during visits. All events will be recorded in the case report form, including their severity, onset and duration. Discontinuation owing to AEs is permitted at any time, and reasons will be documented. Severe or concerning symptoms will prompt immediate contact with the study team. This structured monitoring ensures that both subjective complaints and objective safety concerns are consistently captured. The participant timeline and assessment schedule are detailed in figure 1.

### Sample size calculation

As no study has yet defined the minimal clinically important difference for HR reduction in POTS, we consider the results of Stavrakis *et al*^20^ to be clinically relevant and have therefore used them as the basis for our sample size calculation. In addition, in the absence of direct clinical data on the effect of slow breathing training on POTS, we assumed an additional reduction from slow breathing training based on studies showing that slow breathing training can reduce HR by approximately 4–10 bpm in various cardiovascular conditions.^34 39 40^ We have used a conservative estimate of seven beats per minute, which we believe to be a minimal clinical difference. This reduction represents not only a measurable physiological change but may also contribute to psychological well-being, as studies suggest.^41^ For our clinical trial investigating the effects of AVNT with and without breathing training and a sham AVNT intervention, we calculated the required sample size based on expected HR changes (ΔHR). This calculation was performed for an analysis of variance using HR data from Stavrakis *et al*^20^ and incorporating an assumed reduction of 7 bpm from slow breathing. Although Stavrakis *et al*^20^ reported no significant reduction in HR in the sham AVNT group, we conservatively assumed a placebo-related reduction of 2 bpm for both sham conditions to account for potential expectancy effects.

We estimated the following mean ΔHR values across the four groups:

- AVNT with NB: 17.6 bpm (as reported by Stavrakis *et al*)^20^
- AVNT with slow-paced breathing: 10.6 bpm (17.6–7 bpm)
- Sham AVNT with slow-paced breathing: 22.7 bpm (placebo-adjusted 2 bpm reduction from 31.7 and 7 bpm reduction from slow-paced breathing)
- Sham AVNT with NB: 29.7 bpm (placebo-adjusted 2 bpm reduction from 31.7 bpm)

Assuming an SD of 14 bpm, an alpha level of 0.0167 (Bonferroni correction for 3 pairwise comparisons across four groups), and a power of 80%, 17 participants per group are required for sufficient statistical power. To account for an anticipated 30% dropout rate, 25 participants per group will be recruited, resulting in a total sample size of 100 participants.

Sample size was computed using the University of Vienna’s online analysis of variance (ANOVA) calculator: https://homepage.univie.ac.at/robin.ristl/samplesize.php?test=anova

### Recruitment

We are recruiting participants for our study through various channels. First, we are working closely with support groups such as Long COVID Austria (https://longcovidaustria.at/). We are also using online platforms like POTS-Dysautonomia.net to reach individuals actively seeking information and support for POTS. Additionally, we collaborate with Dr. Michael Stingl, recognised as one of Austria’s leading experts on postacute infection syndrome. Through his numerous TV and radio appearances, he is an important advocate for people with POTS and has an extensive network within the healthcare community. His strong media presence and expertise significantly enhance our ability to identify potential POTS patients.

To ensure successful recruitment for this RCT, we intend to implement the following measures:

1. Recruitment tracking and strategy adjustment

- We will maintain detailed records of all individuals screened, including those deemed eligible and enrolled.
- A monthly review of recruitment progress will be conducted to identify and address any challenges early and make any necessary adjustments to our strategy.
- Recruitment sources will be continuously analysed (eg, online platforms, physician referrals) to identify the most effective channels and allocate resources accordingly.
- If necessary, we will refine our recruitment methods and eligibility criteria to ensure that we reach the target population for the study.

1. Thorough eligibility assessment

- We will systematically document reasons for participant ineligibility to identify common exclusion factors and refine our recruitment strategy as necessary.
- Regular review of the inclusion and exclusion criteria will ensure that they are appropriately representative of the target study population.

1. Participant engagement:

- We will ensure clear and transparent communication of study expectations to build trust and encourage participant engagement.
- Regular check-ins will be scheduled to maintain participant engagement throughout the study.
- We will proactively address any participant concerns or questions to minimise attrition.

1. Feasibility report:

- At the end of the recruitment phase, we will produce a detailed report summarising successful strategies, challenges encountered and key lessons learnt.
- This information will be used to optimise recruitment strategies for future clinical trials in POTS or similar populations.

### Randomisation and blinding

#### Sequence generation

The random allocation sequence will be generated using the Good Clinical Practice (GCP)-compliant, web-based Randomizer service provided by the Medical University of Vienna. This system uses a computer-generated randomisation algorithm to ensure allocation by chance and adherence to GCP standards.

#### Type of randomisation

Stratified block randomisation with a 1:1:1:1 allocation ratio will be used across the four study groups: AVNT+SDB, AVNT+NB, sham AVNT+SDB and sham AVNT+NB. Randomisation will be stratified by PEM status (yes/no), resulting in two strata. The precise block sizes and restrictions are programmed within the Randomiser system but remain undisclosed to the investigators to minimise predictability.

Sex distribution will be monitored throughout recruitment and reported descriptively. Given the expected limited number of male participants, additional stratification by sex was not implemented, as this could result in small and unstable subgroups. However, sex will be considered in the statistical analysis as a covariate or explored in sensitivity analyses, where sample size permits.

#### Allocation concealment mechanism

Allocation concealment is maintained by the central Randomiser system. Group assignment remains hidden until a participant is enrolled and confirmed in the database. All randomisation processes are automatically logged and stored as an electronic audit trail, ensuring complete transparency and traceability.

#### Implementation

The study nurse will be responsible for enrolling participants and entering the necessary data into the Randomiser system. Once randomisation is complete, the group allocation will be retrieved. The PhD student/study nurse and the physiotherapist supervising the breathing therapy will be informed of the participant’s allocation to administer the assigned intervention according to protocol. The statistician responsible for outcome analysis will remain blinded to group allocation until the database is locked.

#### Blinded parties

Participants will remain blinded regarding their AVNT group assignment (active vs sham) by using identical Nurosym devices with standard electrode placement. In the sham condition, the device employs a ‘sound-sham’ method, delivering pulsed acoustic signals that mimic the auditory cues of active AVNT but do not induce physiological vagal activation. These measures ensure participants cannot reliably distinguish between active and sham AVNT. Participants cannot be blinded to the breathing interventions, as they must consciously perform either slow deep breathing or NB techniques. The study nurse, responsible for conducting physiological measurements such as tilt testing and administering questionnaires, cannot be blinded to the intervention group, as she also supervises daily AVNT sessions and directly observes the breathing exercises. To reduce bias, all data will be coded, and the data analyst (statistician) will remain blinded to group allocations until the primary analyses are complete. Unblinded personnel thus include the physiotherapist delivering the breathing training and the study nurse supervising daily AVNT sessions. Outcome assessors and data analysts will remain blinded.

#### Unblinding procedures

Unblinding will only occur if medically necessary, for example, in the event of a SAE where knowledge of treatment allocation is essential for participant safety. Any unblinding will be documented, including the reason, time and person responsible. At the conclusion of the trial, participants from all study arms will be asked to indicate via questionnaire which intervention they believe they received and their level of certainty. This assessment will allow evaluation of the success of blinding.

### Data collection methods

All data will be collected following standardised operating procedures to ensure validity, reproducibility and compliance with GCP. Physiological assessments, including tilt testing, ECG, BP, RSA and capnography, will be conducted by a trained study nurse in accordance with the predefined test protocol. The study nurse will receive specific training in autonomic testing and laboratory safety before the trial begins to ensure consistent assessment practices. Patient-reported outcomes will be gathered using validated and widely recognised questionnaires: the NQ, BPAT, MAPS, SF-12, CFS, HADS. Questionnaires will be provided either in paper format or digitally, depending on participant preference and health condition. To improve data quality, the study nurse will perform immediate plausibility checks of recorded measurements and questionnaires after each visit. All data will be documented in electronic case report forms via REDCap. Range checks and automatic validation rules will be implemented to minimise data entry errors. Paper forms will be available as a backup in case of system failure and will be double-entered into REDCap as soon as possible.

### Plans to promote participant retention and follow-up

During the 12-week intervention, the study nurse will maintain regular contact with participants through phone calls at weeks 2, 6 and 10, as well as email reminders. Participants will also receive text messages or emails 48–72 hours prior to each scheduled visit. If an appointment is missed, an immediate phone call will be made to reschedule and record the reason for non-attendance. Participants who discontinue the intervention will be encouraged to continue participating in follow-up assessments to collect primary and secondary outcome data. If on-site testing is not possible, questionnaire and safety data will be gathered via phone or online survey. All reasons for discontinuation will be documented. The intention-to-treat analysis will include participants with partial outcome data, where feasible.

### Data management

All trial data will be entered into REDCap, a GCP-compliant, password-secured electronic database hosted on secure institutional servers at the Medical University of Vienna. Access will be role-restricted (study nurse, PI, statistician) and all activities will be logged with an audit trail. Data will be anonymised by assigning unique participant identifiers; personal identifiers will be stored separately from trial data. Quality control will include range checks, consistency checks and double data entry for critical variables. Weekly backups will be performed automatically on institutional servers. Only authorised study team members will have access to the final dataset. After study completion, both electronic and paper records will be archived for 15 years in accordance with Austrian General Data Protection Regulation (GDPR).

### Statistical methods

Statistical analyses will be conducted using IBM SPSS Statistics version 28.0 for Windows (IBM). Continuous variables will be expressed as mean±SD deviation (SD), while categorical variables will be shown as frequencies and percentages with 95% CIs. The primary endpoint, HR change (ΔHR), defined as the difference between upright and supine HR, will be analysed using repeated-measures ANOVA with Bonferroni correction applied to account for multiple comparisons. The main comparison will focus on the change in ΔHR from baseline to week 12 across the four intervention groups. Exploratory secondary analyses will examine changes in BP, HRV, RSA, handgrip strength and patient-reported outcomes (MAPS, CFS, SF-12, HADS). For these outcomes, linear regression models incorporating baseline values, age, sex and medication as covariates will be employed. Interaction terms for time-by-group will be included to identify potential differences in trajectories over time. Assumptions of normality and homoscedasticity will be assessed with QQ-plots and residual plots. When appropriate, non-parametric methods will be used for sensitivity analyses. Analyses will follow the intention-to-treat principle, including all randomised participants according to their allocated intervention group, regardless of adherence or protocol deviations. An additional per-protocol analysis will be performed as a sensitivity check, limited to participants with ≥80% adherence to the intervention protocol. Missing data will be addressed through multiple imputation under the assumption of missing at random. Sensitivity analyses will also be conducted using last observation carried forward and complete-case analysis to evaluate robustness. Regarding the results, study nurses will systematically document reasons for missing data in the case report forms. All confirmatory tests for the primary outcome will be two-sided, with a significance level set at α=0.05, adjusted for multiple comparisons using Bonferroni correction. P values from secondary analyses will be considered exploratory and interpreted accordingly.

## Discussion

To our knowledge, this protocol represents the first controlled study to investigate the combined effects of AVNT and SDB in patients with POTS, employing a 2×2 factorial design with four treatment arms to assess both main effects and interaction effects between interventions. This research directly addresses a crucial gap in evidence-based, multimodal management of POTS.

The factorial design will assess whether combining vagal neuromodulation with respiratory training yields superior outcomes compared with each intervention individually. AVNT has already demonstrated promise in a sham-controlled, double-blind RCT, where it significantly reduced orthostatic tachycardia and improved autonomic markers compared with sham stimulation over a 2-month period 21. Similarly, even brief slow deep abdominal breathing at six breaths/min has been shown to significantly reduce standing HR and improve symptom scores in POTS patients during tilt testing.^34^ If additive or synergistic effects are observed, this combined approach may offer a scalable, patient-autonomous and low-risk therapeutic strategy.

Our trial combines objective measures such as HRV, BP responses and validated patient-reported outcomes, providing a comprehensive and multidimensional assessment. The inclusion of a sound-sham AVNT control reduces expectancy bias, while the 12-week intervention period balances therapeutic exposure with practicality for this vulnerable population. Beyond its clinical relevance, this study establishes the groundwork for multimodal autonomic therapy in POTS. The factorial design enables the identification of responder profiles and supports the development of stratified treatment approaches, pushing the field towards precision medicine in autonomic disorders.

Should the combined intervention demonstrate superior efficacy, findings will inform clinical guidelines and healthcare policy regarding non-pharmacological POTS interventions. The ability to deliver both therapies through structured patient training offers potential for scalable, cost-effective treatment that enhances patient autonomy while reducing long-term healthcare burden.

## Conclusions

While non-invasive vagus nerve stimulation and slow-paced breathing have each shown promise in modulating autonomic function, evidence remains limited and fragmented. To our knowledge, our trial is the first to systematically evaluate their combined application in POTS using a rigorous factorial design. By addressing POTS heterogeneity through comprehensive outcome assessment and employing robust sham controls, this study has the potential to clarify the therapeutic role of integrated autonomic modulation. If successful, it will provide the foundation for incorporating neuromodulation and breathing interventions into multimodal, precision-guided care models for this disabling disorder.

### Monitoring

### Data monitoring committee

For this single-centre, investigator-initiated trial, a formal, independent Data Monitoring Committee (DMC) will not be established. The interventions, including auricular vagal neuromodulation and SDB, are considered low-risk and have established safety profiles. The principal investigator will conduct continuous monitoring of trial safety, adherence and data quality in close collaboration with the study nurse and the dedicated clinical trial physician. All AEs will be systematically documented and reported to the Ethics Committee of the Medical University of Vienna in accordance with GCP requirements. Due to the low-risk profile and relatively short trial duration (12 weeks), no interim analyses or formal stopping guidelines are planned. However, if unexpected safety concerns arise, the principal investigator retains full authority, in consultation with the ethics committee, to terminate the trial prematurely.

### Trial monitoring

There will be no independent external monitoring due to the low-risk nature of the trial. Instead, internal monitoring will be carried out by the principal investigator and the study nurses. This includes verifying informed consent, checking for protocol adherence and ensuring data quality. Recruitment logs, adherence records and AE reports will be reviewed at scheduled intervals. A complete audit trail will be kept in REDCap and in the MedUni Vienna Randomizer system to guarantee transparency and traceability of all trial activities.

## Ethics and dissemination

This study has been reviewed and approved by the Ethics Committee of the Medical University of Vienna (EK number 1270/2024). The trial will be conducted in accordance with the Declaration of Helsinki,^42^ the International Conference on Harmonisation Guidelines for GCP and local regulatory requirements.

Written informed consent will be obtained from all participants prior to enrolment. Participation is voluntary, and participants may withdraw at any time without consequences. All data will be pseudonymised and handled in compliance with the GDPR. Identifiable information will be stored separately and securely. Any protocol amendments will be submitted to the ethics committee for approval prior to implementation. On study completion, participants will receive trial results in plain language. Additionally, these results will be shared with healthcare professionals and the scientific community via peer-reviewed publications and conference presentations. The study’s findings will also be reported in the ClinicalTrials.gov registry (NCT06996314) as per regulatory requirements.

### Protocol amendments

Any significant changes to the protocol, including alterations to eligibility criteria, outcomes or statistical analyses, will be submitted as amendments to the ethics committee for approval before implementation. All relevant stakeholders, including the sponsor, investigators and study team members, will be promptly notified of these amendments.

### Consent or assent

Informed consent will be obtained by a trained study nurse, who is also a doctoral student within the project, under the supervision of the principal investigator. Potential participants will receive detailed verbal and written information about the study’s objectives, procedures, risks and benefits. Adequate time will be given for questions before signing the informed consent form. Participants will be told that their participation is voluntary and that they can withdraw at any time without incurring any disadvantage or affecting their ongoing medical care or therapy.

### Confidentiality

Participant confidentiality will be strictly protected. All data will be pseudonymised on collection, using a unique study identification number. Identifiable information, such as names and contact details, will be stored separately from study data in password-protected files accessible only to authorised study nurses and the principal investigator. Electronic data will be stored securely on institutional servers at the Medical University of Vienna, with access restricted to the study team. Data handling adheres to the European Union GDPR.

### Data sharing

De-identified individual participant data, including the data dictionary and the statistical code used for the analyses, will be made available to the corresponding author on a reasonable request after the main results are published. The data will be shared in accordance with institutional and ethical guidelines, ensuring participant confidentiality.
